# Apabetalone, a Clinical-Stage, Selective BET Inhibitor, Opposes DUX4 Target Gene Expression in Primary Human FSHD Muscle Cells

**DOI:** 10.3390/biomedicines11102683

**Published:** 2023-09-30

**Authors:** Christopher D. Sarsons, Dean Gilham, Laura M. Tsujikawa, Sylwia Wasiak, Li Fu, Brooke D. Rakai, Stephanie C. Stotz, Agostina Carestia, Michael Sweeney, Ewelina Kulikowski

**Affiliations:** 1 Resverlogix Corp., 300, 4820 Richard Road SW, Calgary, AB T3E 6L1, Canada; 2 Resverlogix Corp., 535 Mission St., 14th Floor, San Francisco, CA 94105, USA

**Keywords:** facioscapulohumeral muscular dystrophy, FSHD, DUX4, bromodomain and extra-terminal domain, BET inhibitor, transcriptome, RNA, apabetalone, epigenetics

## Abstract

Facioscapulohumeral dystrophy (FSHD) is a muscle disease caused by inappropriate expression of the double homeobox 4 (DUX4) gene in skeletal muscle, and its downstream activation of pro-apoptotic transcriptional programs. Inhibitors of *DUX4* expression have the potential to treat FSHD. Apabetalone is a clinical-stage bromodomain and extra-terminal (BET) inhibitor, selective for the second bromodomain on BET proteins. Using primary human skeletal muscle cells from FSHD type 1 patients, we evaluated apabetalone for its ability to counter DUX4′s deleterious effects and compared it with the pan-BET inhibitor JQ1, and the p38 MAPK inhibitor—and DUX4 transcriptional repressor—losmapimod. We applied RNA-sequencing and bioinformatic analysis to detect treatment-associated impacts on the transcriptome of these cells. Apabetalone inhibited the expression of DUX4 downstream markers, reversing hallmarks of FSHD gene expression in differentiated muscle cells. JQ1, but not apabetalone, was found to induce apoptosis. While both BET inhibitors modestly impacted differentiation marker expression, they did not affect myotube fusion. Losmapimod also reduced expression of DUX4 target genes but differed in its impact on FSHD-associated pathways. These findings demonstrate that apabetalone inhibits DUX4 target gene expression and reverses transcriptional programs that contribute to FSHD pathology, making this drug a promising candidate therapeutic for FSHD.

## 1. Introduction

Facioscapulohumeral muscular dystrophy (FSHD) is a muscular dystrophy characterized by the progressive weakening and atrophy of muscle groups primarily in, but not restricted to, the face (*facio*), around the shoulder blades (*scapula*), and in the upper arms (*humeral*) [1]. This incurable, genetic disease is one of the most prevalent muscular dystrophies, afflicting an estimated 12/100,000 people [2]. Though numerous strategies have been developed to manage symptoms, there are currently no approved treatments for FSHD.

FSHD is caused by the aberrant expression of the embryonic transcription factor double homeobox 4 (*DUX4*) gene in adult skeletal muscle tissue [3]. *DUX4* is located within a D4Z4 macrosatellite repeat array in the subtelomeric region of chromosome 4q35 and in a homologous repeat array on chromosome 10q26 [4]. In healthy, differentiated somatic cells, including muscle cells, the D4Z4 macrosatellite repeat array epigenetically represses *DUX4* expression. Loss of effective repression, attributable to one of several genomic events, permits aberrant *DUX4* transcription and the progressive development of FSHD types 1 and 2 [3]. FSHD type 1 (FSHD1), which accounts for about 95% of cases, results from pathogenically permissive contraction of D4Z4 repeats on chromosome 4 [5]. Patients with FSHD type 2 (FSHD2) have deleterious mutations in epigenetic regulators of the D4Z4 region [6,7]. Though the mechanism of *DUX4* de-repression differs in FSHD1 and FSHD2 genotypes, both alterations cause *DUX4* misexpression in adult muscle tissue. Ultimately, DUX4 activates multiple downstream factors that drive FSHD pathogenesis [8].

Extensive work has been undertaken to understand and characterize DUX4-mediated toxicity in muscle cells [8,9,10,11,12,13,14,15,16,17,18,19]. Although aberrant *DUX4* expression in mature muscle is associated with FSHD [3], it can be difficult to detect even in affected tissues and cells, due to its sporadic expression [20]. Instead, *DUX4* gene expression and protein abundance is typically measured indirectly, using reporter complexes [9] or by quantifying expression of characteristic downstream proteins, such as Zinc Finger and SCAN Domain-Containing Protein 4 (*ZSCAN4*) and Methyl-CpG Binding Domain Protein 3 Like Protein 2 (*MBD3L2*) [8]. Expression of these genes is downstream of DUX4 transcriptional activity, but their individual contributions to FSHD pathology are not yet fully understood. The DUX4 protein is present at elevated levels in differentiated myotubes (fused, mature, multinucleated muscle cells) when compared to undifferentiated myoblasts (muscle progenitor cells) [21]. During differentiation, *DUX4* expression is occasionally, and unpredictably, activated in affected cells [22]. One DUX4-positive nucleus can be sufficient to result in the death of an entire myotube. DUX4-mediated toxicity is driven by the activation of pathways including; tumor suppressor protein P53 (*p53*) signaling [18], signaling associated with the proto-oncogene *MYC* [23], and the suppression of the paired box protein 7 (*PAX7*) pathway [19]. PAX7, in particular, has been found to play a critical role in FSHD pathophysiology [24]. A transcription factor highly expressed in muscle progenitor cells, PAX7 plays a critical role in muscle cell development [25], and drives muscle tissue recovery and regeneration following injury [26]. DUX4 is reported to compete with PAX7 in murine iC2C12 myoblasts with induced *DUX4* expression [27], thereby inhibiting its vital function in preventing muscle damage and atrophy.

As aberrant *DUX4* expression is a causal factor in FSHD pathology, targeting transcriptional regulation of *DUX4* may yield a therapeutic intervention for FSHD. To this end, small molecule inhibitors of glycogen synthase 3-beta (GSK3β) [13], p38 mitogen-activated protein kinases (MAPKs) [28,29], and bromodomain and extra-terminal (BET) proteins [30] have been reported to inhibit *DUX4*. At the most advanced development stage, the p38α inhibitor, losmapimod, is undergoing Phase 3 clinical evaluation for the treatment of FSHD. BET inhibitors (BETi) are particularly interesting FSHD therapeutics as they epigenetically downregulate *DUX4* and the expression of pro-inflammatory genes [31] that exacerbate the condition. Both pan and bromodomain2-selective BETi inhibit *DUX4* expression in FSHD patient derived muscle cells [30].

BETs are evolutionary conserved proteins that play key functions in chromatin organization and regulation of gene transcription. They contain two bromodomains (BD1 and BD2), which bind to acetylated lysines on histones and transcription factors [32]. Bound BET proteins stabilize higher order chromatin structure and provide a scaffold for transcriptional machinery complexes [33]. Of note, depletion of the BET proteins BRD2 and BRD4 with specific short interfering RNAs inhibits *DUX4* expression in FSHD1 myoblasts [30], and BRD2 knockdown represses full length DUX4 protein levels [34]. BETi treatment causes BET proteins to disassociate from acetylated lysines, destabilizing transcriptional complexes at the chromatin, and inhibiting the transcription of proximal genes [35]. Pan-BETi, like JQ1, bind to both BD1 and BD2 with equal affinity, while BD1- and BD2-selective BETi preferentially bind to one or the other [36]. Apabetalone is a BD2-selective BETi [33] currently in advanced clinical development for the treatment of multiple diseases such as cardiovascular disease [37], pulmonary arterial hypertension [38], and COVID-19 [39], with a track record of well-tolerated chronic administration. This contrasts with clinical findings of pan-BETi, which are associated with adverse events (AE) and toxicity that limit their applications primarily to oncology indications [33,40].

In this report, we investigated the effect of apabetalone on the transcriptome of primary human skeletal muscle cells (pHSMCs) from clinically affected FSHD patients. Using RNA-sequencing (RNA-seq) we demonstrated that apabetalone contributes to reversing transcriptional programs that are hallmarks of FSHD. Specifically, we showed that apabetalone reduces the abundance of characteristic downstream target genes of *DUX4* in a dose-dependent fashion. While we observed modest BETi-induced inhibition of muscle cell differentiation markers at higher concentrations, these changes in gene expression did not translate to reduced myotube fusion. Further, we found that apabetalone did not impact pHSMCs’ viability or apoptosis. Our data indicates that the BD2-selective BET inhibitor apabetalone is a strong therapeutic candidate for the treatment of FSHD.

## 2. Materials and Methods

### 2.1. Reagents

Reagents were obtained from Thermo Fisher Scientific (Waltham, MA, USA) unless specified otherwise. Apabetalone and JQ1 were synthesized by NAEJA Pharmaceuticals (Edmonton, AB, Canada) or IRIX Pharmaceuticals (Florence, SC, USA). Selective p38 inhibitor losmapimod was purchased from Selleck Chemicals (Houston, TX, USA).

### 2.2. Cell Culture

Primary human myoblasts were obtained from NIGMS Human Genetic Cell Repository at the Coriell Institute for Medical Research (CIMR; Camden, NJ, USA). Myoblasts were obtained from two clinically affected FSHD1 donors. Donor details are found in Table S1. Donor 1 cells were used for most experiments conducted in this study, due to their rapid growth, and robust DUX4 marker expression. Donor 2 cells were utilized to confirm BETi treatment effects on key markers. Both myoblast isolates were previously characterized, including their expression of myogenic markers, their differentiation time course, as well as their expression of *DUX4* and associated genes [41]. The myogenic purity of the cultures was confirmed by immunofluorescence (IF), using a desmin antibody (ab32362; Abcam plc, Cambridge, UK). We found that isolates from both Donor 1 and Donor 2 consisted of greater than 95% desmin positive cells. The cells were grown on gelatin coated flasks in Ham’s F10 medium—supplemented with 10% fetal bovine serum, penicillin–streptomycin, and 20 ng/mL of human, recombinant, basic fibroblast growth factor (bFGF; Sigma Aldrich, St. Louis, MO, USA). Myoblasts between passage 3 and 11 were used in experiments. Confluent myoblasts were differentiated into myotubes in Skeletal Muscle Differentiation Medium (PromoCell, Heidelberg, Germany) as described (below). Microscopy images were acquired using a Leica (Wetzlar, Germany) DM IL microscope, with a 10×/0.25 objective lens, and a Leica DFC320 camera. DMSO (0.1%) was used as a vehicle for all treatment samples and controls.

### 2.3. mRNA Expression Quantification by Real-Time PCR

Relative mRNA expression was determined by TaqMan based real-time PCR, according to established protocols [39]. Adherent cells, seeded for a minimum of 24 hours (h) were lysed in situ, and RNA was isolated using Catcher PLUS kits (Life Technologies, Waltham, MA, USA). Real-time PCR was used to determine the abundance of each transcript relative to the endogenous control cyclophilin A (gene symbol *PPIA*) using the RNA Ultrasense One-step qRT-PCR kit (Life Technologies). Data was acquired using a ViiA-7 Real-Time PCR apparatus (Applied Biosystems Waltham, MA, USA/Life Technologies). Analysis was performed as 2^(CT cyclophilin–CT marker)^ and results were normalized to DMSO treated samples, as specified in figures. TaqMan assay (Life Technologies) ID numbers are listed in Table S2. Dose–response curves were prepared using an eight-point dilution scale ranging from 0.02–50 µM for apabetalone, and from 0.001–3.0 µM for JQ1.

### 2.4. Immunofluorescence

For IF, cells were fixed with 4% paraformaldehyde in phosphate buffered saline (PBS) for 15 min, permeabilized with 0.2% Triton X-100/PBS for 4 min and blocked with 5% bovine serum albumin (BSA) and 0.02% Triton X-100 in PBS for 30 min at room temperature (RT). Cells were labeled with primary antibody against myosin heavy chain (MHC) (MF20, MAB4470; R&D Systems, Minneapolis, MN, USA) at RT for 4 h, and with goat α–mouse AlexaFluor647 secondary antibody for 30 min at RT. Samples were mounted using ProLong™ Diamond antifade mountant with DAPI (4′,6-diamidino-2-phenylindole). Images were taken using a Zeiss (Oberkochen, Germany) Axioskop 2 Plus fluorescent microscope, with a 10X/0.30 objective lens, and a Leica DFC420 camera. Subsequent image handling was performed in ImageJ. Myofusion Index (MI) was evaluated as the percentage of nuclei included in MHC-positive (MHC^+^) multinuclear cells and reported relative to vehicle control.

### 2.5. Cell Viability and Apoptosis Assays

Myoblast cells from Donor 1 were plated in 96-well plates for 24 h at sufficient density to achieve a confluent monolayer (~25,000 cells/well). After 24 h in growth media, myoblasts were switched to differentiation media and allowed to differentiate for 6 days. Cells were then treated with apabetalone, JQ1, losmapimod, or DMSO vehicle in differentiation media for 72 h. Cells that received 1 µM staurosporine (Life Technologies) for the final 24 h of the treatment period served as a positive control for apoptosis. Cell viability was assessed using the MTS-based CellTiter 96^®^ AQueous One luminescent cell proliferation assay (Promega; Madison, WI, USA), allowing 1 h for the reaction to proceed. Dose–response curves were generated using the same eight-point dilution scales listed above. Apoptosis was assessed using the Caspase-Glo^®^ 3/7 Assay System (Promega). Luminescent quantification was performed using a BioTek Synergy H4 Hybrid Microplate Reader (Agilent, Santa Clara, CA, USA) for both assays according to manufacturer specifications.

### 2.6. RNA-Sequencing

Primary myoblasts from Donor 1 were seeded in a 6-well plate and allowed to adhere for 24 h prior to differentiation. Growth media was changed to differentiation media, and myoblasts were differentiated into myotubes over the next 6 days. Cells were then treated with apabetalone, JQ1, losmapimod, or DMSO vehicle for 72 h. Undifferentiated myoblast samples were also prepared as controls and administered DMSO vehicle for 24 h. All samples were prepared in triplicate. Total RNA was isolated from myoblasts and myotubes using RNeasy kit (Qiagen, Hilden, Germany). Fluorescent quantification of concentration and stability of isolated RNA was performed using a BioTek Synergy H4 Hybrid Microplate Reader equipped with a Take3 Micro-Volume Plate (Agilent). RNA-seq library preparation, sequencing, and mapping was performed by Novogene Corporation (Pasadena, CA, USA) and Genewiz (Chelmsford, MA, USA). Library preparation used Stranded RNA Prep with PolyA selection (Illumina, San Diego, CA, USA), and sequencing was performed using the Illumina HiSeq platform. Reads were mapped to the human transcriptome using the human genome sequence GRCh38. Mapped read counts were normalized and analyzed in R (v4.2.1, The R Foundation for Statistical Computing, Vienna, Austria) using the DESeq2 package [42]. Genes with a log_2_ fold-change (log_2_FC) less than −1.0 or greater than 1.0 between two conditions, with a false discovery rate less than 0.05, were considered significantly differentially expressed, unless otherwise specified.

### 2.7. Composite Biomarker Calculation

Composite biomarkers were calculated from normalized RNA-seq data, according to methods published by Banerji et al. [19]. Three *DUX4* expression biomarkers were computed based on the mean expression of DUX4 target genes identified in Yao et al. [43], Geng et al. [8], and Choi et al. [14]. The PAX7 activity biomarker is computed as the t statistic from a test comparing the upregulated to downregulated PAX7 target genes within each sample [19]. The composite biomarker units are arbitrary and relative to the samples included in each comparison. DUX4 target gene expression scores correlate directly with the severity of FSHD phenotype (i.e., higher score signals more severe disease), while PAX7 target gene expression score is inversely correlated (i.e., lower score signals more severe disease) [44].

### 2.8. Pathway Analysis

Gene Set Enrichment Analysis (GSEA) [45] was performed to characterize the broad, pathway-scale impacts of differentiation and drug treatment on primary FSHD muscle cells. Transcript abundance data was normalized using the DESeq2 package in R [42] and used as the input for GSEA. Normalized enrichment scores (NES) and statistical significance scores were then calculated for the Molecular Signatures Database (MSigDB) GSEA hallmark gene set (v7.5.1) [46] as well as the Kyoto Encyclopedia of Genes and Genomes (KEGG) pathways gene sets (v7.5.1) [47], comparing differentiated myotubes with control myoblasts, as well as treated myotubes with vehicle controls. Computations were performed via GSEA v4.2.3, downloaded from the Molecular Signatures database (http://www.broadinstitute.org/msigdb, accessed on 4 July 2022). Gene sets were considered significantly up- or downregulated with both a nominal *p*-value of less than 0.05, and a false discovery rate (FDR) *q*-value of less than 0.25.

### 2.9. Statistical Analysis

Statistical analysis for PCR and cell viability data was completed using GraphPad Prism 9.4.0 (Dotmatic, Boston, MA, USA). Comparisons between samples were evaluated by one-way ANOVA with Dunnett’s post-testing adjustment for multiple comparisons or by two-way ANOVA with Bonferroni correction, as specified in the figure captions. Half maximal inhibitory concentrations (IC_50_) were derived from fitting parameters of non-linear regression of the log(concentration) and response data using the variable slope, four parameter IC_50_ equation (GraphPad Prism 9.4.0). RNA-seq data was analyzed in R using the DESeq2 package, which fits data to a negative binomial distribution and applies a Wald test to evaluate the statistical significance of differentially expressed genes [42]. Benjamini–Hochberg adjustments were applied to differentially expressed gene *p*-values. Composite biomarker scores were compared by one-way ANOVA with Dunnett’s post-testing, or unpaired Student’s *t*-test, as specified, in GraphPad Prism 9.4.0. Values are displayed as mean plus/minus standard error of the mean (SEM) or as specified. Statistical significances are indicated on figures based on *p*-value thresholds (* < 0.05, ** < 0.01, *** < 0.001, **** < 0.0001).

## 3. Results

### 3.1. DUX4 Target Gene Expression Increases during Differentiation of Primary FSHD Muscle Cells and Is Countered by Apabetalone Treatment

**Figure 1:**
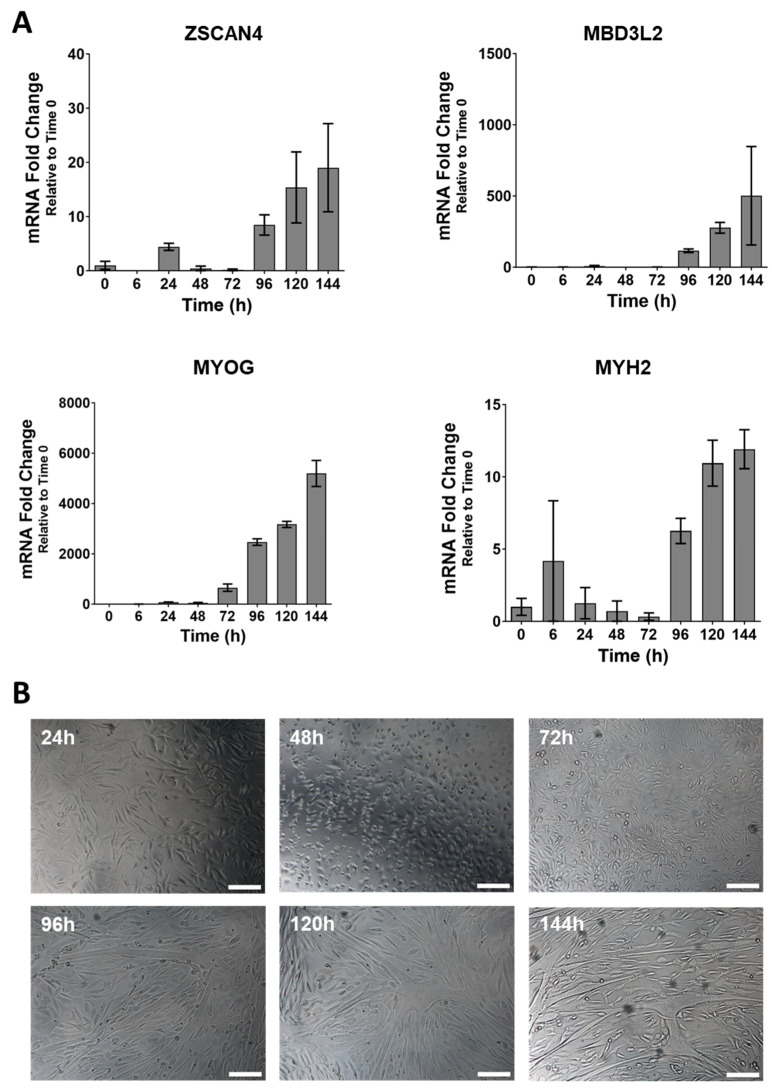
Six-day (144 h) time course of DUX4 and differentiation marker expression in primary human skeletal muscle cells (pHSMCs) from Donor 1 by (**A**) TaqMan based real-time PCR, which demonstrates a marked increase in both DUX4 marker (*ZSCAN4* and *MBD3L2*) and differentiation markers (*MYOG* and *MYH2*) expression beginning after 72 h. Bars show mean ± SEM (standard error of the mean) of two technical replicates at each timepoint, statistical significance not calculated. This timeline is consistent with the appearance of fused myotubes (**B**) in bright field images of pHSMCs from Donor 1 after 72–96 h under differentiating conditions. All images were taken at 10× magnification, scale bars = 100 µm.

Primary skeletal muscle cells from two FSHD1 patients were cultured to evaluate the in vitro impact of apabetalone treatment on DUX4 target gene expression. First, we examined the levels of DUX4 target transcripts during myoblast to myotube differentiation by real-time PCR. Undifferentiated myoblasts expressed low levels of muscle differentiation transcripts myogenin (*MYOG*) and myosin heavy chain 2 (*MYH2*), while expression of DUX4 target gene transcripts, such as *ZSCAN4* and *MBD3L2*, was nearly undetectable in the undifferentiated samples. However, the expression of these genes was notably increased following 96 h of differentiation (Figure 1A). Expression of differentiation and DUX4 target genes continued to increase through the 144 h evaluation period. Myotube fusion was also visually evident in brightfield microscopy images after 96 h (Figure 1B), though some proportion of non-fused mononuclear cells remain visible through 144 h. Thus, primary skeletal muscle cells from FSHD1 patients increased transcription of DUX4 target genes as myoblasts differentiated into myotubes. The observed expression time course of both myogenic and DUX4 markers matches those in a previously reported characterization of the same pHSMCs by Cruz et al. [41].

**Figure 2:**
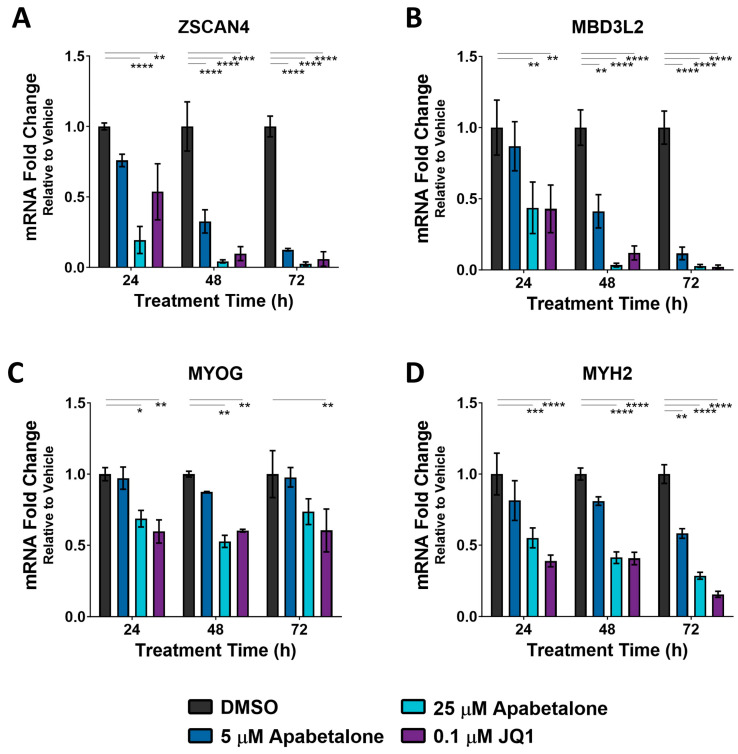
Effects of 24–72 h BET inhibitor treatment times on DUX4 target genes (**A**,**B**) and differentiation markers (**C**,**D**) in differentiated myotubes (six days in differentiation conditions prior to start of treatment) from Donor 1. Bars show mean ± SEM of three technical replicates, significance calculated by two-way analysis of variance (ANOVA) with Bonferroni correction for multiple comparisons. Significance identified by *p*-value threshold (* <0.05, ** <0.01, *** <0.001, **** <0.0001).

We next confirmed that DUX4 target genes were BETi sensitive and determined the optimal BETi treatment time needed for maximal DUX4 target gene inhibition. Myotubes, differentiated for 144 h, were treated with BETi for 24, 48, and 72 h before RNA was harvested and transcript abundance was compared (qRT-PCR). BETi treatment downregulated expression of the DUX4 target genes *ZSCAN4* and *MBD3L2*, which steadily decreased over the course of the treatment (Figure 2A,B). Specifically, following 72 h treatment, apabetalone at 5 µM and at 25 µM, as well as JQ1 at 0.1 µM, all suppressed transcription. In contrast, the transcription of skeletal muscle differentiation markers *MYOG* and *MYH2* was less sensitive to BETi treatment (Figure 2C,D). For instance: 5 µM apabetalone had no impact on *MYOG* expression, irrespective of treatment time, and downregulated *MYH2* by 42% at 72 h, while 25 µM apabetalone and JQ1 significantly reduced both *MYH2* and *MYOG* expression at all three timepoints. Thus, differentiated primary FSHD muscle cells respond to 72 h BETi treatment with a robust, preferential downregulation of DUX4 downstream markers compared to markers of differentiation.

To further evaluate the BETi sensitivity of DUX4 target gene expression in FSHD patient myotubes, dose responses for apabetalone and JQ1 were evaluated (72 h treatment). Apabetalone inhibited the transcription of *ZSCAN4* and *MBD3L2* in Donor 1′s myotubes with IC_50_ values of 1.2 µM and 0.59 µM, respectively (Table 1). The *ZSCAN4* result is in agreement with a previously reported IC_50_ for apabetalone (0.35 µM), in a FSHD2 myoblast cell line [30]. JQ1 was more potent in downregulating these DUX4 target genes based on IC_50_ values. In terms of differentiation markers, apabetalone had little effect on the expression of skeletal muscle differentiation markers *MYOG*, *MYH2*, and *PAX7* (IC_50_ values of 42 µM, 10 µM and 33 µM, respectively, for Donor 1 myotubes) at clinically relevant concentrations (≤5 µM) [33]. Despite potential heterogeneity in primary cells from multiple patients, IC_50_ values for apabetalone on evaluated markers (*ZSCAN4*, *MYOG*, and *MYH2)* were similar between Donor 1 and 2 myotubes (other markers were not evaluated in Donor 2 cells). The pan-BET inhibitor JQ1 robustly inhibited skeletal muscle differentiation marker transcript levels, suggesting a potential role of BET-BD1 in muscle cell differentiation, and raises questions about possible limitations in the therapeutic utility of pan-BETi in FSHD.

### 3.2. Myotube Fusion Is Unaffected by Apabetalone, JQ1, or Losmapimod Treatment

**Figure 3:**
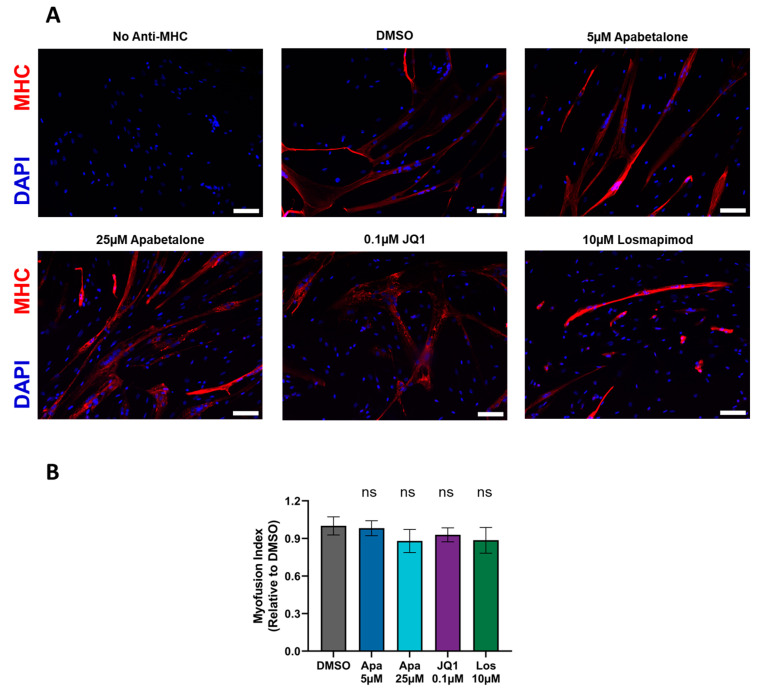
Immunofluorescent images of myosin heavy chain (MHC) and DAPI stained donor 1 myotubes after 120 h of differentiation with labelled treatments applied concurrently to the start of differentiation (**A**). All images were taken at 10× magnification, scale bars = 100 µm. (**B**) MI, defined as the percentage of nuclei within MHC^+^ multinucleated cells, was determined following 120 h of differentiation with concurrent treatment. MHC^+^ and MHC^−^ nuclei counts for three distinct imaging fields were pooled for each sample. Bars show mean ± SEM of three samples, significance calculated by one-way ANOVA with Dunnett’s post-testing for multiple comparisons. ns = not significantly different to DMSO treated cells (*p* > 0.05). Apa = apabetalone; Los = Losmapimod

To assess treatment-induced impacts on muscle cell differentiation, we stained differentiating pHSMCs with anti-MHC and DAPI, and imaged them by fluorescent microscopy (Figure 3A). We observed that after 120 h of differentiation, with treatment starting concurrently with differentiation, the majority of nuclei were found within MHC^+^ multinucleated cells for all treatment conditions. MI, defined as the percentage of nuclei found within MHC^+^ multinucleated cells, was quantified for each treatment condition following 120 h differentiation with concurrent treatment (Figure 3B). The MI is expressed relative to the untreated controls and exhibited no significant impact from any of the applied treatments, compared to the vehicle condition. This evaluation was repeated using the treatment protocol applied most widely in our studies—namely 144 h differentiation, followed by 72 h treatment—which also did not show any treatment impacts on MI (Figure S1). Our differentiation marker expression data and MHC IF data confirm that apabetalone treatment does not negatively impact muscle cell differentiation at concentrations that effectively inhibit DUX4 target genes.

### 3.3. Apabetalone-Mediated DUX4 Target Gene Inhibition Compares Favorably to That of Losmapimod

**Figure 4:**
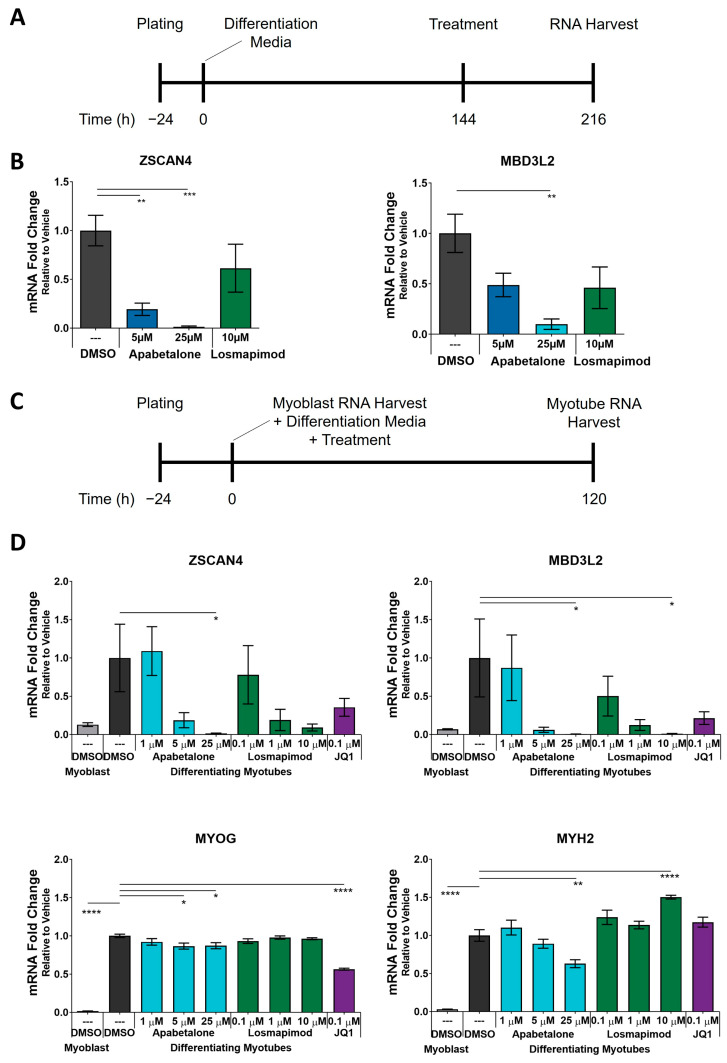
Response to apabetalone and losmapimod treatment was compared using the previously established protocol (**A**) in pHSMCs from Donor 1. DUX4 target gene expression was measured by qRT-PCR (**B**). Bars show mean ± SEM of three technical replicates, statistical significance by one-way ANOVA with Dunnett’s post-testing for multiple comparisons. Failing to capture significant DUX4 target inhibition with 10 µM losmapimod treatment, we applied a parallel differentiation/treatment protocol (**C**) and evaluated treatment impacts of apabetalone and losmapimod on DUX4 target genes (*ZSCAN4* and *MBD3L2*) and differentiation markers (*MYOG* and *MYH2*) (**D**). Bars show mean ± SEM of six technical replicates, and statistical significance was assessed by one-way ANOVA with Dunnett’s post-testing. Significance identified by *p*-value threshold (* <0.05, ** <0.01, *** <0.001, **** <0.0001).

We next compared downregulation of DUX4 target genes by BETi with that of losmapimod. Losmapimod is a p38α/β MAPK inhibitor and a FSHD therapeutic candidate, which has been shown to inhibit *DUX4* expression in multiple FSHD1 and FSHD2 cell lines [29]. Losmapimod was selected as a comparator compound due to its documented inhibition of DUX4 and its advanced clinical development. We first tested losmapimod’s effect on expression of DUX4 target genes in differentiated FSHD myotubes (Figure 4A), where 10 µM losmapimod did not alter expression of DUX4 target genes (Figure 4B). Losmapimod treatment was previously reported to inhibit DUX4 target gene expression at much lower concentrations (*MBD3L2* IC_50_ = 0.03 µM) using a concurrent differentiation/treatment protocol [29], where adherent primary myoblasts were treated with test articles for 120 h in differentiation media (Figure 4C). Under these conditions we found that high concentrations of apabetalone and losmapimod significantly reduced expression of *MBD3L2* (Figure 4D), but only apabetalone significantly reduced *ZSCAN4* expression. Losmapimod treatment did not impact the expression of the skeletal muscle differentiation marker *MYOG*, but significantly increased the abundance of *MYH2* transcripts at 10 µM (Figure 4D). In contrast, BETi treatment (apabetalone and JQ1) had little impact on *MYOG*, while 25 µM apabetalone decreased *MYH2* transcript levels. Overall, these results suggest that BET inhibition can be an effective strategy to counter DUX4 target gene expression in differentiating FSHD myotubes, with comparable effects to high dose losmapimod.

### 3.4. Apabetalone Benefits Individual and Composite Markers of FSHD

RNA-seq was applied to evaluate the impact of apabetalone treatment on the transcriptome of differentiated pHSMCs and compare its effects with those of JQ1 and losmapimod. As previously detailed, myoblasts were differentiated for 144 h prior to treatment initiation, which lasted an additional 72 h. Differential gene expression was evaluated between undifferentiated and differentiated pHSMCs to characterize the impacts of differentiation, as well as the activation of *DUX4* expression during differentiation. Differentiated myotube drug treatment conditions were compared with vehicle control myotubes to evaluate treatment effects.

**Figure 5:**
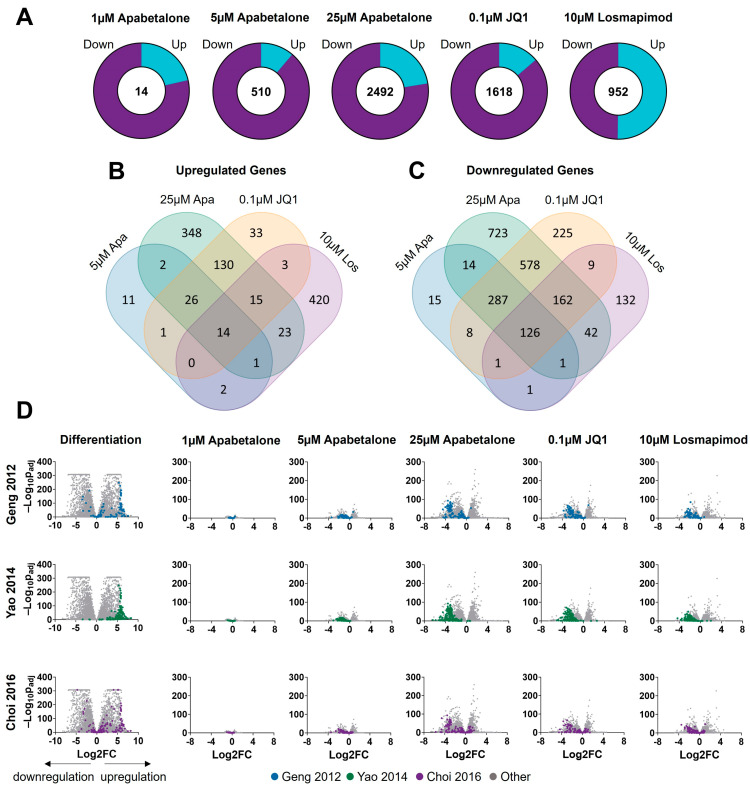
RNA-seq results for apabetalone, JQ1 and losmapimod treatment of differentiated pHSMCs from Donor 1 (**A**). Relative numbers of up- and downregulated genes are shown in the pie charts, while total numbers of differentially expressed genes are shown in the middle. Venn diagrams show the overlap in upregulated (**B**) and downregulated (**C**) genes between different treatment conditions (1 µM apabetalone treatment excluded for clarity and due to relatively few differentially expressed genes). Volcano plots (**D**) highlight the impacts of differentiation and treatment impacts on DUX4-associated markers. Points highlighted in color represent genes reported to be upregulated with DUX4 by Geng et al. [8] (blue), Yao et al. [43] (green), or Choi et al. [14] (purple), as labelled. Grey dots represent all other detected transcripts. Analysis by DESeq2 in R, all samples evaluated in triplicate.

Apabetalone-mediated effects on the myotubes’ transcriptomes were dose dependent. The number of differentially expressed genes compared to the vehicle control increased with increasing concentration of apabetalone, from 14 at 1 µM to 2492 at 25 µM (Figure 5A). The number of differentially expressed genes with JQ1 (0.1 µM) and losmapimod (10 µM) treatment were 1618 and 952, respectively. Most impacted genes were downregulated for each of the BETi treated conditions (78–89% down) (Figure 5A), whereas approximately half of losmapimod-affected genes were upregulated. The overlap in differentially expressed genes between BETi-treated myotubes and losmapimod-treated myotubes was much more significant for the downregulated genes than for upregulated genes. A total of 126 differentially expressed genes were downregulated with apabetalone (5 µM and 25 µM), JQ1, and losmapimod treatment (Figure 5C), whereas only 14 were upregulated by all four conditions (Figure 5B). The vast majority (88%) of differentially expressed genes upregulated with losmapimod treatment were unique to that condition alone, while 72% of losmapimod’s significantly downregulated genes were shared with at least one BET inhibitor.

Next, we examined the effect of apabetalone, JQ1 and losmapimod treatment on FSHD makers of disease. Among the downregulated genes shared by BETi and losmapimod were some well-known downstream markers of *DUX4* expression (*ZSCAN4*, *MBD3L2*, etc.). DUX4′s transcriptional program—overlapping sets of hundreds of genes associated with DUX4′s transcriptional activity and characterized in Geng et al. [8], Yao et al. [43], and Choi et al. [14] in various model systems of FSHD—was highly activated during differentiation of pHSMCs. Volcano plots (Figure 5D) highlight genes upregulated by DUX4 in these three published datasets (indicated by corresponding-colored datapoints, grey datapoints represent all other detected transcripts). Most members—73%, 69%, and 58%, respectively—of all three gene sets were significantly upregulated (log_2_FC > 0, p_adj_ < 0.05) during differentiation in our system (left column; Figure 5D). Differentiation-induced upregulation of DUX4 target genes was countered (log_2_FC < 0) by apabetalone treatment (subsequent three columns of volcano plots) in a dose-dependent fashion with many markers reaching significant downregulation in the 5 µM condition. DUX4-mediated gene expression was also countered with 25 µM apabetalone, JQ1 and losmapimod.

**Figure 6:**
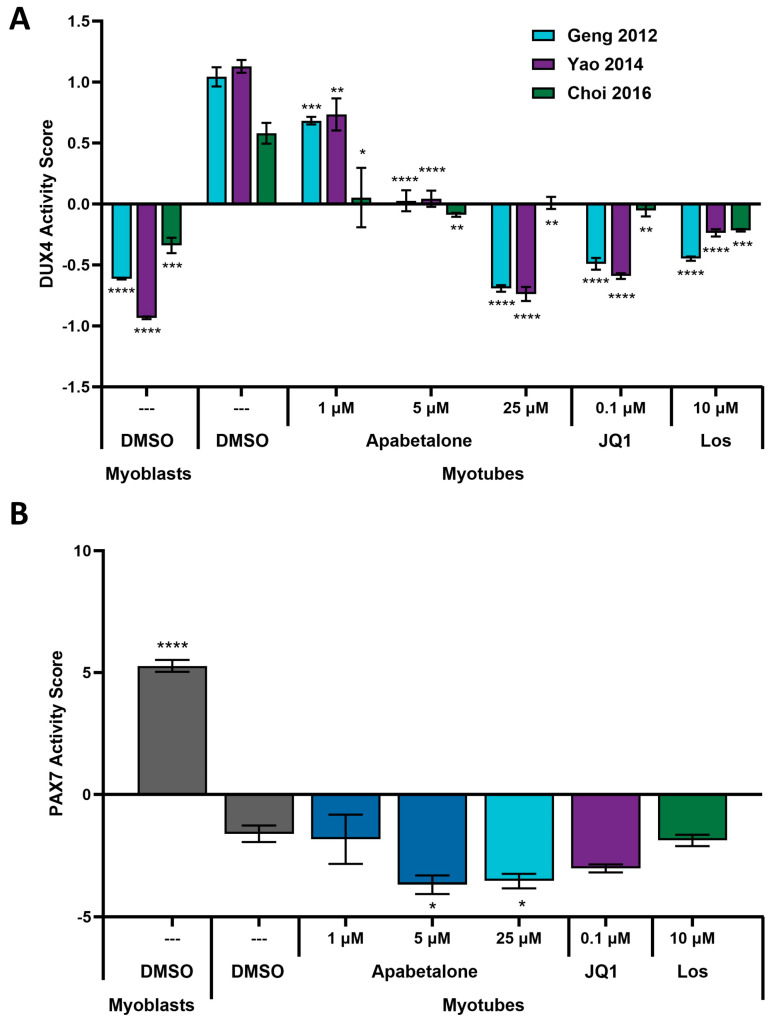
Composite biomarkers of DUX4 (**A**) and PAX7 (**B**) activity, derived and validated by Banerji et al. [19,44], were used to evaluate treatment impact on pHSMCs based on RNA-seq data. DUX4 and PAX7 activity scores for each condition are relative to the other conditions in this comparison, and the units are arbitrary. Bars show mean ± SEM of three technical replicates, and statistical significance was assessed by one-way ANOVA with Dunnett’s post-testing and shown relative to vehicle (DMSO) control myotubes. Significance identified by *p*-value threshold (* <0.05, ** <0.01, *** <0.001, **** <0.0001).

Using the three gene sets referenced above, and methods developed by Banerji et al. [19], we computed the expression of composite biomarkers to evaluate overall DUX4 activity (listed in Table S4). These scores represent the average, normalized expression of all markers in each published gene set and provide a single, validated metric of DUX4 activity [44]. As expected, DUX4 signature gene expression significantly increased for all three composite biomarkers with activation of DUX4 during pHSMC differentiation (Figure 6A; DMSO myoblasts vs. DMSO myotubes). Next, we compared the effect of BETi versus losmapimod on these computed DUX4 activity scores. Apabetalone treatment at clinically relevant doses (1 µM and 5 µM) countered this DUX4 activity signal, while apabetalone at high dose of 25 µM abolished this induced activity at a comparable level to JQ1 and 10 µM losmapimod (Figure 6A). PAX7 expression scores were also calculated for each control and treated condition, using the composite markers developed by Banerji et al. [19], as summarized in Table S5. PAX7 expression and its downstream genes are inversely correlated with DUX4 expression in FSHD muscle cells [17], and PAX7 activity score is associated with FSHD pathology and disease progression [44]. PAX7 activity was reduced during differentiation and was further decreased by apabetalone treatment (5 µM and 25 µM; Figure 6B). The relatively long differentiation time may factor into the limited treatment impact on PAX7 score, as PAX7 is more abundant in less differentiated pHSMCs. This hypothesis is supported by the significant increase in PAX7 score observed with 5 µM apabetalone treatment in intermediate differentiated pHSMCs (72 h of differentiation, followed by 24 h of treatment: (Figure S2A)). Apabetalone treatment also did not impact PAX7 activity in undifferentiated myoblasts (Figure S2B).

Impacts on terminal markers of skeletal muscle differentiation, previously compiled and published by Chal et al. [48], are shown in Table S3. Notably, apabetalone’s observed downregulation of *MYH2* expression by qPCR (Figure 2E) did not generalize to other late myogenesis markers in our RNA-seq data. Myosin heavy chain genes *MYH3*, *MYH7*, and *MYH8* were significantly upregulated (FC > 1, p_adj_ < 0.05) by all three evaluated concentrations of apabetalone, while *MYH1* was upregulated by 1 and 5 µM concentrations. Of the 21 analyzed genes, only two: Myogenic Factor 6 (*MYF6*) and Calcium Voltage-Gated Channel Subunit Alpha1 H (*CACNA1H*) were significantly downregulated (FC < 1, p_adj_ < 0.05) by 5 µM apabetalone, and none at 1 µM (Table S3). Broader, but still inconsistent, downregulation of differentiation markers was observed with 25 µM apabetalone (9/21), JQ1 (4/21), and losmapimod (9/21) treatment. We also examined post-synaptic markers of neuromuscular junction (NMJ) function [49], and dystrophin-associated complex (DAC) genes [50] (Table S4). All evaluated treatment conditions had a mixed impact on these markers, significantly upregulating and downregulating some of these markers. As with the terminal differentiation markers, broader downregulation of NMJ and DAC markers were observed with 25 µM apabetalone (14/33) and JQ1 (14/33), than with 1 µM (1/33) or 5 µM (7/33) apabetalone. Overall, apabetalone treatment did not result in a broad negative impact on late myogenesis, NMJ function, or DAC markers in our RNA-seq evaluation.

### 3.5. BET Inhibition and p38 Inhibition Have Different Impacts on DUX4-linked Pathways

Pathway analysis of the RNA-seq data for differentiated and treated pHSMCs was conducted using GSEA [45], and referencing the MSigDB Hallmarks gene set [51] as well as KEGG pathways [47]. Rickard et al. [9] took advantage of the stochastic nature of *DUX4* expression to isolate DUX4′s transcriptomic effects on FSHD myoblasts. First, they used a DUX4-activated reporter to isolate the *DUX4*-expressing subset of cells from a population of FSHD myoblasts, then, using RNA-seq, they compared the transcriptome of the DUX4-posivitive cells with DUX4-negative cells from the same population, and identified differentially regulated pathways. We examined these DUX4-impacted KEGG pathways to see the effects of BETi and losmapimod treatment. NES and FDR values for pathways significantly activated or deactivated in our experiments are shown in Table 2 (significantly regulated pathways in Rickard et al. with no significant changes in our study are excluded to simplify the table). NES was applied to evaluate the enrichment each pathway’s member genes in the treated conditions compared to vehicle control samples (positive NES values indicate enrichment in the treated samples relative to the controls, while negative values indicate the opposite), while FDR assessed statistical significance in those enrichments. Treatment with apabetalone resulted in the significant upregulation of five pathways (at 25 µM), four of which were identified by Rickard et al. as downregulated in *DUX4* expressing cells (Lysosome, Glutathione Metabolism, Other Glycan Degradation, GnRH Signaling Pathway) and the other was bidirectionally disrupted (Endocytosis), indicating that apabetalone reverses DUX4-mediated pathway activity. JQ1 significantly upregulated six pathways, all of which were downregulated by DUX4 (Apoptosis, Lysosome, Glutathione Metabolism, Other Glycan Degradation, p53 Signaling Pathway, GnRH Signaling Pathway). As expected, there was clear overlap between JQ1 and apabetalone treatment. In contrast, there was no overlap between BET inhibitor treatment and p38 inhibition by losmapimod. Losmapimod treatment resulted in six significantly downregulated pathways; two upregulated by DUX4 (Spliceosome, Basal Transcription Factors), three downregulated by DUX4 (Focal Adhesion, Gap Junction, Vascular Smooth Muscle Contraction), and one bidirectionally disrupted pathway (Adherens Junction). Thus, BET inhibition effectively counters dysregulation in a subset of DUX4-associated KEGG pathways, while p38 inhibition impacts a completely distinct subset of these pathways, countering some and exacerbating others.

**Figure 7:**
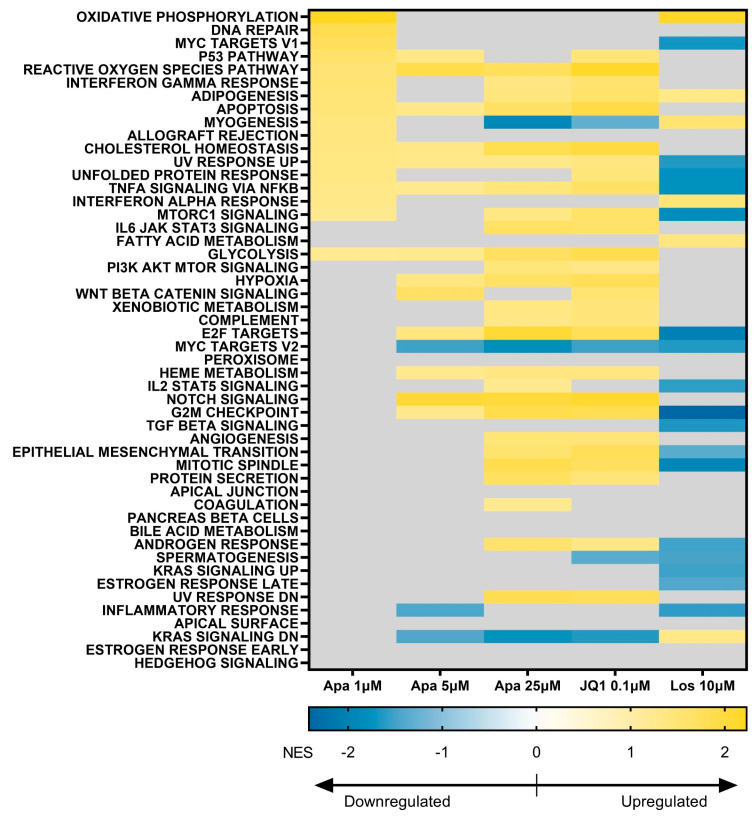
Heatmap showing myotube treatment effects on MSigDB Hallmark gene sets, based on normalized RNA-seq transcript abundances. Normalization by DESeq2 package in R and computations were performed via GSEA (v4.2.3). Color scale for each cell represents NES value, shown for statistically significantly regulated pathways only (*p* < 0.05 and FDR < 0.25). Negative NES values (blue) show downregulated pathways in treated conditions relative to the vehicle, while positive NES values (yellow) indicate that the pathways are regulated by treatment.

MSigDB Hallmarks is a set of 50 pathways that represent well-defined biological states or processes [46], a number of which are applicable to muscle cells and/or are associated with DUX4 activity. We analyzed changes in these pathways with pHSMC differentiation and in each of our treatment groups (Figure 7). Myogenesis was significantly upregulated by 1 µM apabetalone and 10 µM losmapimod, not significantly affected by 5 µM apabetalone, and significantly downregulated by 25 µM apabetalone treatment and by JQ1. Several pathways with documented links to DUX4 activity and FSHD pathology were significantly impacted by one or more treatments: p53 Pathway [18], MYC Targets V1 [23], MYC Targets V2 [23], Inflammatory Response [16], DNA Repair [52], Reactive Oxygen Species Pathway [52], WNT Beta Catenin Signaling [13], and Apoptosis [8,23]. These findings are also consistent in at least two cases (p53 Pathway, Apoptosis) with differentiation effects on relevant KEGG pathways (Table 2). BET inhibitor and losmapimod treatment demonstrated shared responses in some relevant pathways, including: MYC Targets V2 and Inflammatory Response, which were both significantly downregulated with losmapimod treatment and at least one BET inhibitor condition. Other important pathways, including: p53 Pathway, MYC Targets V1, Reactive Oxygen Species Pathway, Apoptosis, WNT Beta Catenin Signaling, and DNA Repair were differentially regulated between BET inhibition and p38 inhibition. The exact role of these pathways in FSHD is unclear, however they have been linked to DUX4 activity and pathology.

### 3.6. Cell Viability and Apoptosis in pHSMCs Are Not Negatively Impacted with Apabetalone Treatment

**Figure 8:**
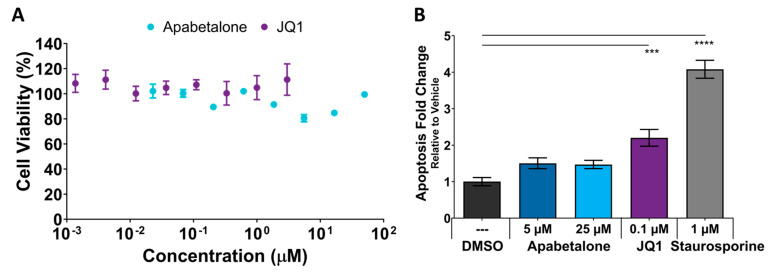
Apabetalone and JQ1 have no impact on pHSMC viability as assessed by a colorimetric MTS assay normalized to vehicle (DMSO) control (**A**). Cells from Donor 1 were differentiated for 144 h, followed by 72 h BET inhibitor treatment. Plotted values represent mean ± SEM of six technical replicates. Apoptosis was assessed by caspase 3/7 assay (**B**) following the same differentiation and treatment protocol and normalized to the vehicle control. Positive control wells were treated with staurosporine for one day prior to assessment. Values represent mean ± SEM of three technical replicates. Statistical significance was assessed by one-way ANOVA with Dunnett’s post-testing, relative to the vehicle control. Significance identified by *p*-value threshold (*** <0.001, **** <0.0001).

The impact of BETi treatment on differentiated FSHD myotube cell viability and apoptosis were evaluated. Myoblasts were differentiated for six days prior to three-days of test compound treatment (or one day for staurosporine positive control wells). Cell viability was assessed using an MTS-based kit. Neither apabetalone nor JQ1 significantly reduced the viability of the cells at any of the evaluated concentrations (Figure 8A). Apoptosis was evaluated by measuring Caspase 3/7 activity [53]. Apabetalone had no significant impact on Caspase 3/7 activity levels, even at higher concentrations, but JQ1 treatment increased activity by more than 2-fold (Figure 8B). Thus, mild pro-apoptotic effects were associated with JQ1 that did not alter cell viability.

## 4. Discussion

Aberrant *DUX4* expression is the root cause of muscle degeneration in FSHD patients. Repression of *DUX4* transcription is therefore an attractive therapeutic target for halting FSHD disease progression. Here we show that apabetalone downregulates expression of DUX4 target genes, improves composite biomarkers of DUX4 activity, counters downregulation of some DUX4-associated KEGG pathways, and improves some MSigDB Hallmarks linked to FSHD pathology. Apabetalone is a well-studied therapeutic candidate, with a history of chronic administration in other indications. Across numerous clinical trials [37,54], over 1900 subjects have received apabetalone for up to three years, totaling more than 3000 patient-years of exposure. These clinical trials demonstrate that apabetalone is well tolerated and that AE are mild, with little discernible difference between placebo- and active-treated subjects. Notably, in these cardiovascular disease populations, patients randomized to apabetalone reported fewer instances of muscle complaints than those randomized to placebo [37]. Our findings suggest that apabetalone treatment may be able to reduce DUX4-associated pathology in FSHD patients and, combined with its established clinical safety and tolerability data, apabetalone is a promising clinical candidate for FSHD.

Previously, it has been reported that DUX4 activates a broad transcription program [8,43] known to be pro-apoptotic in muscle cells, however tracing the particular contribution of individual factors or pathways to this outcome has proved challenging [15,18,19,23]. Hence, the upstream targeting of DUX4 expression itself has been suggested as the best therapeutic strategy. Significant work has been completed to understand the epigenetic mechanism underlying *DUX4* misexpression in FSHD1 and FSHD2 patients [3,6,7,20,21,43], to identify druggable targets, as well as pharmacologic candidates [13,23,28,29,30]. One promising avenue of inquiry has been the epigenetic regulation of *DUX4* gene expression through inhibition of BET family proteins. Here, we confirm and expand upon the work of Campbell et al. [30], who first reported the inhibition of *DUX4* and its downstream markers by BET protein inhibitors. In their study, the authors found that apabetalone (RVX-208) inhibited *ZSCAN4* in immortalized myoblasts (MB200) derived from an FSHD2 patient. Our studies, in differentiated primary myotubes from two FSHD1 patients, yielded notably similar results, given the differences in cells and methodology.

We built on these findings by using RNA-seq to take a broader view of apabetalone’s impact on the transcriptome of FSHD muscle cells. Apabetalone treatment reduced the expression of the majority of DUX4 target genes, suggesting a broad repression of DUX4′s transcriptional program. This was quantified using DUX4 and PAX7 composite biomarkers, developed and validated by Banerji et al. [19,44], which have been correlated with disease severity and progression. BETi treatment significantly inhibits DUX4 activity in these cells, but did not rescue the PAX7 activity score—an interesting result given their previously reported association [24]. We speculate that this may be the result of low endogenous PAX7 in terminally differentiated muscle cells, and at earlier timepoints—where PAX7 is more abundant [25]—apabetalone either improved or had no impact on PAX7 scores.

Apabetalone’s transcriptional effects went beyond direct DUX4 target genes; BET inhibition also upregulated DUX4-downregulated KEGG pathways related to metabolism and cell death [9], which have clear roles in FSHD pathology. For example, apabetalone counters DUX4-associated downregulation of Glutathione Metabolism (glutathione is a powerful regulator of reactive oxygen species), which contributes to elevated oxidative stress susceptibility in FSHD muscle cells [27,55]. Analysis of MSigDB Hallmark pathways also revealed apabetalone upregulated pathways with the potential to benefit FSHD cells, such as DNA Repair, an important process to counter DUX4-induced damage [52], and WNT Beta Catenin Signaling, previously found to prevent DUX4-associated apoptosis [13]. In combination, these findings demonstrate robust, apabetalone-induced repression of DUX4 activity, and mediation of its negative impacts.

Our study further examined the differences between apabetalone at clinically relevant and BD2-selective inhibition concentrations (≤5 µM) and pan-BETi (JQ1 and apabetalone at higher concentrations >20 µM) [33]. A much broader spectrum of genes can be affected by pan-BET inhibition than by BD2-selective inhibition, contributing to differential treatment outcomes [31,56]. In our experiments, treatment with 0.1 µM JQ1 or 25 µM apabetalone resulted in considerably more differentially expressed genes than treatment with apabetalone at a BD2-selective 5 µM concentration. Inhibition of DUX4 target gene expression was evident with both apabetalone and JQ1, suggesting *DUX4* inhibition does not require BD1 binding. BRD4 has a central role in the maintenance of higher-order chromatin structure [33], and pan-BETi can cause alterations to fundamental cellular programming, resulting in potentially deleterious impacts on proliferation and cell survival signaling. This was evidenced by the significant increase in apoptosis (caspase 3/7) seen with JQ1, but not apabetalone. While pan-BET inhibition is associated with chromatin decondensation and fragmentation, BD2-selective BETi alter gene expression without disrupting chromatin.

We also evaluated losmapimod, a p38 inhibitor undergoing clinical evaluation for the treatment of FSHD. Although previous work found that p38 inhibition significantly inhibited *DUX4* transcription and potently reduced expression of DUX4 target genes [28,29], we found that losmapimod was less responsive in differentiated myotubes. Losmapimod did demonstrate more robust nominal inhibition of DUX4 target genes using a parallel differentiation/treatment protocol, but statistical significance was not achieved at lower concentrations, likely due to noise deriving from less robust marker expression under these conditions. Rojas et al. [29] reported few differentially expressed genes with losmapimod treatment in FSHD myotubes beyond DUX4 target genes. We found 952 differentially expressed genes with 10 µM losmapimod treatment, the majority of which were not linked to DUX4. There is significant overlap in downregulated genes between BET inhibitor and losmapimod treated pHSMCs, but very little overlap in upregulated genes. While both BETi and losmapimod had significant impacts on KEGG pathways associated with DUX4 in pHSMCs, there was little overlap in the pathways differentially regulated by BET inhibitors and by losmapimod. Those affected by losmapimod treatment were related to transcription and intercellular adhesion. Losmapimod countered DUX4-associated upregulation of transcription related pathways, but it further downregulated intercellular adhesion pathways that were already reduced by DUX4. It is not clear what impact this would have on disease biology. These findings show that both BET inhibition and p38 inhibition have broader impacts beyond repressing DUX4 and each class benefited different aspects of the DUX4-induced transcriptome.

As *DUX4* expression is dependent on, and occurs in tandem with, myotube differentiation, our findings do not disentangle pathway effects resulting from *DUX4* expression with those resulting from differentiation. This is a limitation of our study. Other work has overcome this issue by employing inducible *DUX4* expression systems, which allow direct transcriptomic comparison of the same cells with and without *DUX4* [55]. Unfortunately, these systems are inappropriate for the evaluation of BET inhibitors due to the epigenetic mechanism of action of these compounds. Models with non-endogenous *DUX4* have *DUX4* transcription occurring outside of its native epigenetic environment, with transcriptional complexes that may be entirely different from those driving *DUX4* expression in pHSMCs from FSHD patients. These induced-expression systems also lack chromatin structure, which, as Tsujikawa et al. recently highlighted [33], helps define the set of genes and pathways that are impacted by BETi. Another important limitation to our study is the sample size. Our work included pHSMCs from only two donors, almost all of our experiments were conducted in Donor 1 cells. *DUX4* expression and transcriptional activation of DUX4 target genes can vary significantly among FSHD cell samples [43]. The generalizability of our findings to other FSHD cells is not assured.

Previously, Roberts et al. [57] reported the essential role for BET proteins in skeletal muscle differentiation, and the negative impact of pan-BETi (JQ1) on the differentiation of C2C12 mouse skeletal muscle cells. Our studies did not yield a significant impact of JQ1 on myofusion, however concentrations used in our studies were toward the low end of their reported range. We found that apabetalone treatment had little to no impact on differentiation markers at clinically relevant concentrations, but modestly inhibited *MYOG* and *MYH2* at higher concentrations. As with JQ1, apabetalone’s inhibition of differentiation marker gene expression did not translate to impacts on myofusion, even at 25 µM. Looking at a broader array of terminal differentiation markers by RNA-seq revealed mixed effects with some markers upregulated and some downregulated by treatment. Myogenesis Hallmark pathway showed a dose-dependent response to apabetalone—upregulated at 1 µM, unaffected at 5 µM, and downregulated at 25 µM. Importantly, most of our experiments were conducted in myotubes that were under differentiation conditions for 144 h prior to treatment. It is unclear what effect, if any, decreased expression of these markers would have on the cells. When we evaluated apabetalone and JQ1 using a concurrent differentiation/treatment protocol we observed smaller nominal differences in relative *MYOG* and *MYH2* expression, despite a longer (120 h) treatment time. Overall, our findings suggest it is unlikely that apabetalone would negatively impact muscle tissue repair and regeneration at clinically relevant concentrations.

## 5. Conclusions

*DUX4* transcription, the underlying cause of FSHD development and progression, is sensitive to BET protein inhibition. Here we demonstrate that the BD2-selective BET inhibitor, apabetalone, inhibits DUX4 target gene expression and reverses the transcriptional program that it activates. Furthermore, we show the differentiated impacts on measures of muscle cell health and function between BD2-selective and pan-BET inhibitors. These findings suggest that apabetalone, as the only BET inhibitor tolerated for chronic administration, has a strong potential for the treatment of FSHD.

## Figures and Tables

**Table 1:** BETi dose–response for DUX4 target genes and myotube differentiation markers.

Gene of Interest		Donor 1		Donor 2	
		Apabetalone IC_50_ (µM)	JQ1 IC_50_ (µM)	Apabetalone IC_50_ (µM)	JQ1 IC_50_ (µM)
DUX4 Targets	*ZSCAN4*	1.2	0.014	0.69	0.011
	*MBD3L2*	0.59	0.015	nd	nd
Differentiation	*MYOG*	42	0.28	>50	0.47
	*MYH2*	10	0.033	>50	0.12
	*PAX7*	33	>3.0	nd	nd

Optimized 72 h treatment time was applied to dose–response assays evaluating apabetalone and JQ1 treatment in differentiated Donor 1 and Donor 2 myotubes. IC_50_ values were derived from fitting parameters of non-linear regression of log(concentration) versus response data using a variable slope IC_50_ equation (GraphPad Prism 9.4.0). IC_50_ values were derived from 3–5 technical replicates (nd = no data).

**Table 2:** Differentiation and Treatment Impacts on DUX4-linked Pathways.

	Apabetalone						JQ1		Losmapimod	
	1 µM		5 µM		25 µM		0.1 µM		10 µM	
	NES	FDR	NES	FDR	NES	FDR	NES	FDR	NES	FDR
DUX4 Upregulated Pathways (Rickard 2015 [9])										
Spliceosome	ns		ns		ns		ns		−1.96	0.02
Basal Transcription Factors	ns		ns		ns		ns		−1.86	0.04
DUX4 Downregulated Pathways (Rickard 2015 [9])										
Focal Adhesion	ns		ns		ns		ns		−1.36	0.23
Apoptosis	ns		ns		ns		1.31	0.21	ns	
Lysosome	1.73	0.02	1.92	0.02	2.62	<0.001	2.46	<0.001	ns	
Glutathione Metabolism	1.36	0.23	ns		1.68	0.04	1.62	0.09	ns	
Gap Junction	ns		ns		ns		ns		−1.49	0.23
Vascular Smooth Muscle Contraction	ns		ns		ns		ns		−1.36	0.24
Other Glycan Degradation	ns		ns		1.84	0.02	1.57	0.08	ns	
p53 Signaling Pathway	ns		ns				1.42	0.15	ns	
GnRH Signaling Pathway	ns		ns		1.26	0.23	1.26	0.24	ns	
DUX4 Bidirectionally Disrupted Pathways (Rickard 2015 [9])										
Endocytosis	ns		ns		1.30	0.23	ns		ns	
Adherens Junction	ns		ns		ns		ns		−1.55	0.17

Normalized enrichment score (NES) and false discovery rate (FDR) values for KEGG pathways which were differentially regulated by DUX4 [9] and at least one treatment condition in our dataset. Sorting of upregulated, downregulated, and bidirectionally disrupted pathways based on DUX4-induced response observed by Rickard et al. [9]. Positive NES values indicated upregulation of pathways, while negative values corresponded to downregulation. Pathways with no significance (*p* < 0.05 and FDR < 0.25) were indicated with ns.

## Data Availability

The datasets used and/or analyzed during the current study are available from the corresponding author on reasonable request.

## Supplementary Materials

The following supporting information can be downloaded at: https://www.mdpi.com/article/10.3390/biomedicines11102683/s1, Figure S1: Myofusion index was not impacted by apabetalone, JQ1, or losmapimod treatment; Figure S2: Apabetalone treatment effects on PAX7 activity score depend on stage of differentiation; Table S1: Donor details and source of pHSMCs; Table S2: List of TaqMan assay IDs used for qRT-PCR evaluation of pHSMCs; Table S3: Impact of differentiation and treatment on terminal differentiation markers by RNA-seq in Donor 1 Cells; Table S4: Impact of differentiation and treatment on muscle function associated markers by RNA-seq in Donor 1 cells; Table S5: Composition and derivation of composite FSHD transcriptomic biomarkers.
