# Supplementary material for: Apabetalone, a Clinical-Stage, Selective BET Inhibitor, Opposes DUX4 Target Gene Expression in Primary Human FSHD Muscle Cells
Source: Biomedicines. 2023 Sep 30;11(10):2683. doi: 10.3390/biomedicines11102683 (PMC10604783; doi:10.3390/biomedicines11102683)
Supplement: Supplementary file 1 [file biomedicines-11-02683-s001.zip › biomedicines-2562277-supplementary.pdf]

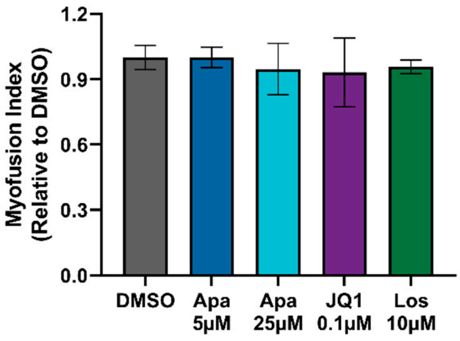

Figure S1: Myofusion Index was Not Impacted by Apabetalone, JQ1, or Losmapimod Treatment

Myofusion Index (MI), defined as the percentage of nuclei within myosin heavy chain (MHC) positive multinucleated cells, was determined following 144h of pre-differentiation and 72h of treatment. MHC^+ and MHC^- nuclei counts for 3 distinct imaging fields were pooled for each sample. Bars show mean ± SEM of three samples, significance calculated by one-way ANOVA with Dunnett's post-testing for multiple comparisons.

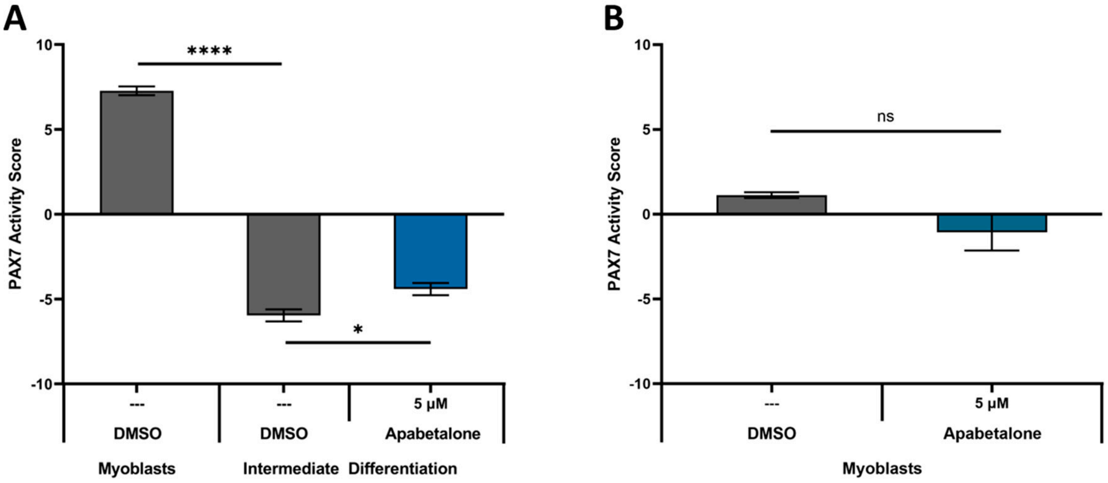

Figure S2: Apabetalone Treatment Impacts on PAX7 Activity Score Depend on Stage of Differentiation

Composite biomarkers of and PAX7 activity, derived and validated by Banerji et al. [19,44], were used to evaluate treatment impact on undifferentiated (A) pHSMCs, and intermediate differentiation (B) based on RNA-seq data. Intermediate samples were allowed to differentiate for three days prior to treatment and all samples were treated for one day. Bars show mean ± SEM of three technical replicates, and statistical significance was assessed by one-way ANOVA with Dunnett's post-testing for intermediate samples and by unpaired Student's t-test for undifferentiated samples.

Table S1: Donor Details and Source of pHSMCs

**Cell ID**	**Donor 1** (GM17869)	**Donor 2** (GM17940)
Source	NIGMS Human Genetic Cell Repository	NIGMS Human Genetic Cell Repository
Genotype	FSHD1	FSHD1
Clinically Affected	Yes	Yes
D4Z4 Repeats – 4q35	5 / 31	3 / 33
D4Z4 Repeats – 10q26	5 / 39	15 / 26
Age at Sampling	21	13
Sex	Female	Male
Cell Type	Skeletal myoblast	Skeletal myoblast

Table S2: List of TaqMan Assay IDs Used for qRT-PCR Evaluation of pHSMCs

**Gene Name**	**Symbol**	**TaqMan Assay ID**
Zinc Finger and SCAN Domain-Containing Protein 4	*ZSCAN4*	Hs00537549_m1
Methyl-CpG Binding Domain Protein 3 Like Protein 2	*MBD3L2*	Hs00544743_m1
Myosin Heavy Chain 2	*MYH2*	Hs01126511_m1
Paired Box 7	*PAX7*	Hs00242962_m1
Myogenin	*MYOG*	Hs01072232_m1
Cyclophilin A	*PPIA*	Hs99999904_m1

Table S3: Impact of Differentiation and Treatment on Terminal Differentiation Markers by RNA-seq in Donor 1 Cells

**Terminal Skeletal Muscle Differential Markers (Chal 2017 [48])**		**Differentiation**		**Apabetalone**						**JQ1**		**Losmapimod**	
		**Vehicle**		**1 µM**		**5 µM**		**25 µM**		**0.1 µM**		**10 µM**	
		**FC**	**padj**	**FC**	**padj**	**FC**	**padj**	**FC**	**padj**	**FC**	**padj**	**FC**	**padj**
**Muscle Regulatory Factors**													
Myogenic Differentiation 1	MYOD1	**1.21**	**8E-03**	**1.37**	**2E-04**	**1.36**	**5E-05**	**1.35**	**3E-05**	**1.31**	**3E-04**	0.99	0.95
Myogenin	MYOG	**40.76**	**<1E-308**	**1.31**	**4E-03**	**1.21**	**0.04**	0.91	0.34	1.09	0.40	1.02	0.85
Myogenic Factor 6	MYF6	**2.18**	**1E-09**	0.88	0.46	**0.54**	**9E-06**	**0.33**	**3E-16**	**0.50**	**1E-07**	1.13	0.49
Nuclear Factor I X	NFIX	1.02	0.79	0.98	0.79	**1.16**	**0.02**	**1.27**	**2E-05**	**1.34**	**7E-08**	**1.40**	**9E-10**
**Primary Myogenesis Markers**													
Myosin Heavy Chain 3	MYH3	**39.30**	**<1E-308**	**1.22**	**5E-04**	**1.35**	**2E-10**	**1.13**	**0.01**	**1.37**	**1E-11**	**0.76**	**5E-09**
Myosin Heavy Chain 7	MYH7	**33.27**	**<1E-308**	**1.34**	**2E-03**	**1.58**	**1E-08**	**1.35**	**1E-04**	**1.60**	**1E-09**	**4.21**	**4E-86**
Myosin Heavy Chain 8	MYH8	**36.04**	**<1E-308**	**1.30**	**2E-05**	**1.60**	**3E-19**	**1.34**	**4E-08**	**1.81**	**2E-31**	1.03	0.69
Myosin Light Chain 1	MYL1	**42.26**	**<1E-308**	1.13	0.18	1.00	0.98	**0.58**	**2E-17**	1.00	0.99	**0.84**	**0.02**
Actin Alpha Skeletal Muscle 1	ACTA1	**42.32**	**<1E-308**	1.16	0.18	1.08	0.52	0.85	0.09	1.05	0.70	**0.71**	**1E-04**
Actin Alpha Cardiac Muscle 1	ACTC1	**17.55**	**<1E-308**	**1.43**	**1E-05**	**1.56**	**4E-10**	**1.43**	**2E-07**	**1.56**	**2E-10**	0.92	0.43
Cholinergic Receptor Nicotinic Gamma Subunit	CHRNG	**43.18**	**<1E-308**	0.98	0.84	1.20	0.06	1.15	0.14	1.10	0.40	**0.83**	**0.04**
Calcium Voltage-Gated Channel Subunit Alpha1 H	CACNA1H	**44.87**	**2E-212**	0.97	0.74	**0.82**	**0.01**	**0.29**	**1E-78**	**0.55**	**3E-20**	**0.40**	**7E-44**
Solute Carrier Family 2 Member 1	SLC2A1	**0.17**	**<1E-308**	**1.13**	**0.05**	**1.18**	**2E-03**	**1.44**	**1E-16**	**1.32**	**2E-09**	**0.82**	**4E-05**
Solute Carrier Family 2 Member 4	SLC2A4	**26.70**	**2E-35**	**1.43**	**0.01**	**1.36**	**0.02**	**0.47**	**7E-10**	0.79	0.09	1.17	0.31
**Secondary Myogenesis Markers**													
Myosin Heavy Chain 1	MYH1	**29.30**	**6E-306**	**1.33**	**2E-06**	**1.41**	**1E-10**	**0.82**	**4E-04**	**1.19**	**2E-03**	**0.41**	**2E-62**
Myosin Heavy Chain 2	MYH2	**41.12**	**<1E-308**	0.97	0.78	0.88	0.12	**0.44**	**6E-38**	**0.50**	**7E-26**	**1.22**	**0.01**
Myosin Heavy Chain 4	MYH4	**27.22**	**3E-05**	0.79	nr	1.07	0.90	0.86	0.73	0.93	0.89	1.07	0.90
Myosin Light Chain 3	MYL3	**23.41**	**3E-08**	1.18	nr	1.25	0.58	**0.42**	**5E-03**	0.60	0.12	0.70	0.34
Actin Alpha Skeletal Muscle 1	ACTA1	**42.32**	**<1E-308**	**1.16**	**0.18**	1.08	0.52	0.85	0.09	1.05	0.70	**0.71**	**1E-04**

Cholinergic Receptor Nicotinic Epsilon Subunit	CHRNE	**0.60**	**0.03**	0.82	nr	0.86	0.71	0.87	0.70	0.84	0.63	0.79	0.50
Calcium Voltage-Gated Channel Subunit Alpha1 S	CACNA1S	**41.65**	**<1E-308**	1.02	0.78	1.01	0.93	**0.56**	**7E-38**	**0.77**	**4E-08**	**0.75**	**2E-09**
Calcium Voltage-Gated Channel Subunit Alpha1 C	CACNA1C	0.74	0.05	0.83	0.38	0.90	0.69	1.21	0.31	1.28	0.17	1.10	0.71
Solute Carrier Family 2 Member 4	SLC2A4	**26.70**	**2E-35**	**1.43**	**0.01**	**1.36**	**0.02**	**0.47**	**7E-10**	0.79	0.09	1.17	0.31

Terminal differentiation markers reported in Chal et al. [48]. CACNA1F, CACNA1D, CACNA1G, CACNA1I, SLC2A2 excluded from analysis due to low transcript abundance. nr = not reported in DESeq2 differential expression analysis. FC = fold change, padj = Benjamini-Hochberg adjusted p-value. Significantly differentially expressed markers highlighted in bold (padj <0.05).

.

Table S4: Impact of Differentiation and Treatment on Muscle Function Associated Markers by RNA-seq in Donor 1 Cells

Neuromuscular Junction and Dytrophin-associated Complex Markers		Differentiation		Apabetalone						JQ1		Losmapimod	
		Vehicle		1 µM		5 µM		25 µM		0.1 µM		10 µM	
		FC	padj	FC	padj	FC	padj	FC	padj	FC	padj	FC	padj
**Defined Neuromuscular Junction Function (Rodríguez Cruz 2020 [49])**													
Agrin	AGRN	**1.49**	**2E-14**	1.12	0.10	**1.30**	**3E-06**	**1.47**	**3E-13**	**1.37**	**5E-09**	1.03	0.70
Cholinergic Receptor Nicotinic Alpha Subunit	CHRNA1	**10.46**	**<1E-308**	1.02	0.76	**0.82**	**1E-07**	**0.48**	**5E-103**	**0.67**	**2E-32**	**1.34**	**7E-17**
Cholinergic Receptor Nicotinic Beta Subunit	CHRNB1	**5.89**	**<1E-308**	1.01	0.93	0.95	0.36	**0.83**	**2E-06**	**0.81**	**6E-08**	**1.13**	**3E-03**
Cholinergic Receptor Nicotinic Gamma Subunit	CHRNG	**43.18**	**<1E-308**	0.98	0.84	1.20	0.06	**1.15**	**1E-01**	**1.10**	**4E-01**	**0.83**	**4E-02**
Cholinergic Receptor Nicotinic Delta Subunit	CHRND	**36.78**	**<1E-308**	1.08	0.28	1.06	0.43	1.07	0.32	1.10	0.14	**0.66**	**1E-15**
Cholinergic Receptor Nicotinic Epsilon Subunit	CHRNE	**0.60**	**0.03**	0.82	nr	0.86	0.71	0.87	0.70	0.84	0.63	0.79	0.50
Docking Protein 7	DOK7	**2.56**	**3E-38**	1.06	0.61	0.88	0.18	**0.67**	**4E-08**	**0.71**	**1E-05**	1.03	0.84
LDL Receptor Related Protein 4	LRP4	**8.22**	**5E-112**	0.87	0.27	0.88	0.32	**0.63**	**1E-06**	**0.70**	**3E-04**	**0.62**	**9E-07**
Microtubule Actin Crosslinking Factor 1	MACF1	**2.33**	**2E-14**	0.82	0.20	0.84	0.26	0.82	0.14	0.89	0.46	**0.70**	**0.01**
Muscle Associated Receptor Tyrosine Kinase	MUSK	**8.33**	**5E-58**	0.72	0.05	**0.68**	**0.01**	**0.36**	**1E-15**	**0.51**	**4E-07**	1.04	0.86
Plectin	PLEC	**0.74**	**5E-06**	0.99	0.95	1.15	0.10	**1.37**	**3E-06**	**1.30**	**2E-04**	0.96	0.70
Receptor Associated Protein Of The Synapse	RAPSN	**2.69**	**1E-44**	1.06	0.59	0.85	0.09	**0.52**	**2E-19**	**0.67**	**8E-08**	1.10	0.32
Sodium Voltage-Gated Channel Alpha Subunit 4	SCN4A	**47.01**	**<1E-308**	1.04	0.69	1.02	0.87	**0.76**	**2E-06**	**0.78**	**4E-05**	**1.24**	**8E-05**
ALG2 Alpha-1,3/1,6-Mannosyltransferase	ALG2	**0.63**	**1E-24**	0.98	0.82	1.07	0.35	**1.23**	**4E-05**	1.09	0.15	**0.78**	**4E-06**
ALG14 UDP-N-Acetylglucosaminyltransferase Subunit	ALG14	**0.64**	**1E-05**	0.93	0.66	0.77	0.06	**0.78**	**0.05**	**0.72**	**8E-03**	0.88	0.39
Dolichyl-Phosphate N-Acetylglucosaminephosphotransferase 1	DPAGT1	**0.69**	**4E-10**	1.07	0.46	1.03	0.78	1.02	0.88	0.97	0.77	0.94	0.51

Glutamine--Fructose-6-Phosphate Transaminase 1	GFPT1	**0.75**	**4E-06**	0.90	0.22	1.02	0.90	1.12	0.12	1.10	0.22	**0.85**	**0.03**
GDP-Mannose Pyrophosphorylase B	GMPPB	**0.55**	**2E-17**	1.05	0.64	1.06	0.62	0.93	0.44	0.99	0.94	0.95	0.65
Solute Carrier Family 25 Member 1	SLC25A1	**0.44**	**5E-17**	**1.34**	**0.03**	**1.47**	**7E-04**	**2.09**	**2E-13**	**1.68**	**8E-07**	1.14	0.35
**Dystrophin-associated Complex (Gao 2015 [50])**													
Dystroglycan 1	DAG1	**1.57**	**5E-22**	1.03	0.64	**1.23**	**1E-04**	**1.50**	**7E-18**	**1.45**	**1E-14**	**0.86**	**8E-03**
Dystrophin	DMD	**11.60**	**5E-96**	0.81	0.21	0.98	0.94	0.95	0.81	1.02	0.94	**0.71**	**0.01**
Dystrobrevin Alpha	DTNA	**6.06**	**2E-185**	1.01	0.90	**0.84**	**7E-03**	**0.53**	**5E-30**	**0.78**	**3E-05**	1.09	0.22
Dystrobrevin Beta	DTNB	0.99	0.97	0.90	0.57	1.13	0.51	**1.66**	**2E-05**	1.19	0.28	1.29	0.08
Laminin Subunit Alpha 2	LAMA2	**2.06**	**4E-14**	0.87	0.33	1.02	0.93	1.26	0.03	1.24	0.06	0.84	0.15
Sarcoglycan Alpha	SGCA	**4.32**	**4E-89**	1.19	0.08	1.02	0.88	**0.75**	**3E-04**	**0.81**	**0.02**	**1.28**	**3E-03**
Sarcoglycan Beta	SGCB	0.93	0.26	0.96	0.68	1.13	0.10	**1.42**	**3E-09**	**1.31**	**1E-05**	0.96	0.62
Sarcoglycan Gamma	SGCG	**43.16**	**2E-123**	0.98	0.86	**0.71**	**4E-05**	**0.26**	**6E-60**	**0.48**	**2E-20**	**1.53**	**5E-08**
Sarcoglycan Delta	SGCD	**8.73**	**2E-169**	**0.67**	**1E-05**	**0.49**	**1E-19**	**0.23**	**2E-82**	**0.38**	**7E-36**	**0.63**	**2E-08**
Syntrophin Alpha 1	SNTA1	**0.64**	**3E-10**	1.18	0.09	1.11	0.33	1.16	0.07	1.14	0.15	1.06	0.63
Syntrophin Beta 1	SNTB1	**4.06**	**8E-86**	0.85	0.09	**0.79**	**4E-03**	**0.58**	**2E-13**	**0.71**	**7E-06**	1.10	0.36
Syntrophin Beta 2	SNTB2	**0.30**	**3E-97**	1.03	0.77	**1.26**	**1E-03**	**2.12**	**9E-37**	**1.83**	**4E-23**	1.08	0.39
Sarcospan	SSPN	**11.01**	**<1E-308**	0.92	0.31	**0.78**	**4E-05**	**0.52**	**1E-31**	**0.71**	**2E-09**	0.91	0.23
Utrophin	UTRN	0.77	0.18	0.71	0.16	0.79	0.35	1.01	0.97	1.03	0.94	0.80	0.37

Neuromuscular Junction Function markers reported in Rodríguez Cruz et al. [49] and dystrophin-associated complex marker from Gao et al. [50]. NOS1, SNTG1, and SNTG2 excluded from analysis due to low transcript abundance. nr = not reported in DESeq2 differential expression analysis. FC = fold change, padj = Benjamini-Hochberg adjusted p-value. Significantly differentially expressed markers highlighted in bold (padj <0.05).

Table S5: Composition and Derivation of Composite FSHD Transcriptomic Biomarkers

Source of Gene List	Target	Target-associated Genes	Composite Readout	Method	Derivation Model	Reference
Geng (2012)	DUX4	165 upregulated	Mean of z-score normalized gene expression data	Microarray	DUX4 transduced myocytes	[8]
Yao (2014)	DUX4	213 upregulated	Mean of z-score normalized gene expression data	RNA-seq	DUX4 transduced myocytes, primary muscle cells, and muscle tissue biopsies	[43]
Choi (2016)	DUX4	212 upregulated	Mean of z-score normalized gene expression data	RNA-seq	DUX4 transduced myocytes	[14]
Banerji (2017)	PAX7	311 upregulated<br>290 downregulated	t-statistic of z-score normalized gene expression data	Microarray	PAX7 transduced myocytes	[19]
